# The Importance of Community Consultations for Generating Evidence for Health Reform in Ukraine

**DOI:** 10.15171/ijhpm.2016.104

**Published:** 2016-08-17

**Authors:** Olena Hankivsky, Anna Vorobyova, Anastasiya Salnykova, Setareh Rouhani

**Affiliations:** ^1^London School of Hygiene and Tropical Medicine, London, UK.; ^2^School of Public Policy, Simon Fraser University, Vancouver, BC, Canada.; ^3^Institute for Intersectionality Research and Policy, Simon Fraser University, Vancouver, BC, Canada.; ^4^Institute of Population Health, University of Ottawa, Ottawa, ON, Canada.

**Keywords:** Ukraine, Health Crisis, Community Consultations, Public Perceptions, Citizen Engagement, Civil Participatory Approach, Social Determinants of Health (SDH), Health Reform

## Abstract

**Background:** The paper presents the results of community consultations about the health needs and healthcare experiences of the population of Ukraine. The objective of community consultations is to engage a community in which a research project is studying, and to gauge feedback, criticism and suggestions. It is designed to seek advice or information from participants directly affected by the study subject of interest. The purpose of this study was to collect first-hand perceptions about daily life, health concerns and experiences with the healthcare system. This study provides policy-makers with additional evidence to ensure that health reforms would include a focus not only on health system changes but also social determinants of health (SDH).

**Methods:** The data collection consisted of the 21 community consultations conducted in 2012 in eleven regions of Ukraine in a mix of urban and rural settings. The qualitative data was coded in MAXQDA 11 software and thematic analysis was used as a method of summarizing and interpreting the results.

**Results:** The key findings of this study point out the importance of the SDH in the lives of Ukrainians and how the residents of Ukraine perceive that health inequities and premature mortality are shaped by the circumstances of their daily lives, such as: political and economic instability, environmental pollution, low wages, poor diet, insufficient physical activity, and unsatisfactory state of public services. Study participants repeatedly discussed these conditions as the reasons for the perceived health crisis in Ukraine. The dilapidated state of the healthcare system was discussed as well; high out-of-pocket (OOP) payments and lack of trust in doctors appeared as significant barriers in accessing healthcare services. Additionally, the consultations highlighted the economic and health gaps between residents of rural and urban areas, naming rural populations among the most vulnerable social groups in Ukraine.

**Conclusion:** The study concludes that any meaningful reforms of the health sector in Ukraine must include a broad range of factors, including the healthcare system but importantly, must extend to SDH approach and include the prioritization of health promotion, limiting alcohol and tobacco availability and enforcing environmental protection.

## Background

Over the last decade, increased attention has been paid to the growing population health challenges in former Soviet states (fSU).^1-6^ Recent events, however, have specifically propelled Ukraine, a country that occupies a strategic and critical geographic position in Europe, to the international fore. The crisis in Ukraine that began in the fall of 2014 has been framed as largely political and economic even though it has significant humanitarian, environmental, and health dimensions. Moreover, there is a lack of explicit recognition that events of the last year exacerbate what was already a pre-existing health crisis, marked by significant health inequities.^7^

To date, research on fSU, including Ukraine, has documented the poor health of populations, including patterns and distribution of premature and preventable deaths^8,9^ health behaviours and their underlying causes,^10-13^ psychological distress^14-16^ also and the ineffective state of existing healthcare services and delivery^5,17-19^ and specific burdens of out of pocket (OOP) payments for accessing healthcare.^9,20^

Studies have also examined life satisfaction,^21^ established links between wider socio-economic and political changes and the health of populations.^22-25^ While producing invaluable evidence, leading studies in the field have tended to be epidemiological and quantitative. There are, however, a few noteworthy exceptions. For example, in 2003, a group of researchers undertook qualitative research in three regions of Ukraine: Kherson, Lviv, and Chernobyl.^20,23^ In the 2006^20^ publication, the researchers discussed findings on the population’s understanding of health and the factors influencing it and in the 2010 publication,^23^ they drew on a social quality model to examine conditions for well-being and conditions for building and sustaining societies – the physical and psychological conditions for empowerment for well-being. Other qualitative studies have been more narrowly focused, examining experiences of specific sub-populations.^9,20,21^

In response to the current focus of research, there have been recent calls to extend the evidence base regarding health problems and challenges in fSU^4^ including more studies that capture first-hand perceptions, experiences and self-expressed needs of affected populations. This is in-line with growing international pressures for civil society engagement, through participatory research methods, to inform the development of policy and program intervention.^26-29^ Such processes are crucial for securing wide spread understanding and support for health reform strategies.^30-34^ The resulting information can also inform the development of policies that better meet the needs of the population and lead to empowerment in communities to take ownership of their health and well-being.^31^

The purpose of our study was to contribute to the existing knowledge base of the health crisis in Ukraine. Using the conceptual framework of the World Health Organization (WHO)^25^ which is detailed below, we gathered information about public perceptions of health, the healthcare system and factors related to well-being. More specifically, our research question was to find out the residents’ of Ukraine self-reported perceptions about what affects their quality of life and what factors influence their experiences of health, including their interactions with the healthcare system, as well as what recommendations for healthcare reforms citizens have for the policy-makers. Our research consisted of multi-site, focus group community consultations that took place across eleven regions of Ukraine in both rural and urban settings. We collected qualitative data about the general population’s experiences in relation to the healthcare system, including issues of quality, access and affordability. Our intention was to gather a breadth of information, in a preliminary fashion, about the lives and health of Ukrainians across the nation, in the way of rich narratives and personal experiences. Evidence generated in this manner can inform health reform efforts by placing front and centre the insights of the population whose health and lives are directly affected by persistent and growing inequities.

As with all fSU, slow socio-economic progress, coupled with deteriorating social security and political instability have contributed to high morbidity, lower life expectancy, and growing health inequities within the population; the magnitude of which is only starting to be recognized.^4,35,36^ For example, over 25% of the adult Ukrainian population, between the ages of 18 to 65 years, has a chronic condition or disease.^36^ The average life expectancy at birth is 9.1 years lower than in the European Union (EU). The gender mortality gap is 10 years, with women living much longer than their male counterparts.^37^ The combination of high death and ultra-low fertility rates are projected to lead Ukraine to experience the single largest absolute population loss in Europe between 2011 and 2020.^38^ Current health outcomes, including premature mortality rates among working class males, result in human and economic costs for individuals, their families, and the nation as a whole. At the same time, according to the World Bank, about 50% of deaths under the age of 75 could be prevented with adequate prevention and treatment interventions in Ukraine.^36^

And yet, Ukraine’s healthcare system has remained virtually unchanged from the breakdown of the Soviet Union. Based on the Semashko model of care (inherited from the Soviet Union) local polyclinics are the initial point of entry for primary, specialist care and referrals to hospitals and higher-level services in this model. Efficacy of the health system is measured by the number of hospital beds and medical employees. And although the Constitution of Ukraine (section 49) guarantees that health services should be provided by the government free of charge, in reality, access to care is hindered by substantial OOP payments required to access care. For example, in 2006 OOP expenses accounted for 40.2% of total health expenditure and were second only to the expenditure of local governments at 42% ^[1] ^.^39^ Importantly, the focus of most reform efforts, until very recently, have been on the system itself with very little attention to public health or health promotion.

## Methods

### Conceptual Framework

The study was grounded in the social determinants framework utilized by the WHO Commission on the Social Determinants of Health (CSDH)^25^ to explain underlying processes and causes of health inequities. According to the model, complex relationships exist between social determinants of health, structural causes of health inequities, health systems, and how they interact and affect health outcomes. In accordance with this approach, our questions targeted to address the contextual and underlying causes of poor health in relation to the experiences and circumstances of daily living of individuals that range from individual level to community-based, health system, and societal factors. Also worth highlighting is that little information is available first-hand – and especially in the last decade, from the Ukrainian population itself, on how political, social, and economic forces in combination with health system and access factors shape their lives and health.

### Study Design

The choice of community consultations was modelled after a similar process in Australia where priorities for men’s health policy were formulated with the help of national public consultations^40^ The norm of community inclusion and participation is well-recognized in the global health sector.^28,29^ There is solid evidence that community engagement interventions have a positive impact on health behaviours, health consequences, self-efficacy and perceived social support outcomes, across various conditions.^41^ The purpose of this study was to provide a venue for Ukrainian citizens to discuss what shapes and influences their lives, their health, experiences in seeking healthcare and providing recommendations for policy change.

The consultations, undertaken in Ukraine in 2012 were held in an equal combination of rural and urban settings. The qualitative study involved 21 community consultations in 11 regions of Ukraine including the Autonomous Republic of Crimea, Cherkasy, Dnipropetrovsk, Donetsk, Kherson, Kyiv, Lviv, Sumy, Zaporizhia, Vinnytsia, and Zhytomyr, regions that encompass a diversity of Ukraine’s demographics, culture, and geography (see Appendix A). Participants were recruited by local non-governmental organization (NGO) partners (recognized for their work on community development) from their client networks via posters, newsletters, email, and telephone outreach. Each NGO was tasked with recruiting an equal number of male and female participants of a variety of ages from three broad categories – healthcare consumers, health service providers and local government representatives. Each participant was provided with a modest honorarium of 50 UAH (equivalent of approximately US$7 in 2012). In the end, 844 individuals of diverse genders, ages, and socio-economic statuses participated in the consultations. Each consultation had between 35-50 participants. Female participants accounted for two thirds of those in attendance – not surprising given the gendered divisions in health interest evidenced by literature in the field.^42^

Prior to the field research, the principal investigator (PI) conducted team training to ensure consistency and quality assurance of each consultation. Additionally, with the assistance of the team, the PI produced detailed guidelines to set out the entire process, including steps in the consultations, and roles and responsibilities of all team participants and information about community consultations as a qualitative method. The initial draft of the guide and consultation questions were written in English and then translated (both into Ukrainian and Russian) by one of the research assistants and verified by another research assistant. One NGO partner was also involved in the verification of the translated documents to ensure their easy readability in Ukrainian and Russian.

Each community consultation was overseen by at least two representatives of the research team (eg, PI and research assistants) as well as least three representatives from the supporting NGO organization. Consultations consisted of open-ended discussions in groups of 6-10 people moderated by members of the research team and trained facilitators. The questions discussed at each forum (see Appendix B) were developed by the research team upon the analysis of an earlier quantitative part of the research (Omnibus survey conducted in 2011), literature review and in consultation with the Ministry of Health (MoH).

Gatherings took place in schools, community centres, libraries, museums, halls, or NGO offices. Each forum lasted 2-3 hours and consisted of a brief information session about the purpose of the study followed by discussion in small groups. After an informal coffee-break, key points and recommendations were presented by a participant from each table. Each table discussion was digitally recorded. Students from local universities were also recruited to take notes (in the language of discussion) to ensure manual back up recording.

Ethics approval was received from the ethics committees of all three academic partnering institutions – Simon Fraser University (Burnaby, BC, Canada), The National University of Kyiv-Mohyla Academy (Kiev, Ukraine), and the University of Alabama in Birmingham, AL, USA. Before each consultation, the purpose of the research was presented and the voluntary nature of participation was emphasized. An information letter and consent form were distributed to all participants, in the language of their preference (eg, Ukrainian or Russian) and only those participants that signed the forms participated in the research.

### Data Analysis

All the digitally recorded consultations were professionally transcribed. They were then translated from Russian and/or Ukrainian into English by one translator.

The analysis consisted of identifying and describing themes and patterns using the MAXQDA 11 software. Three researchers were involved in the coding of approximately equal portions of the data. Two of the individuals were present at community consultations as coordinators, note takers or facilitators. Codes were developed by three analysts and the principal investigator in collaboration. The research team used an iterative and data-driven process of creating codes that were organized into themes representing frequently occurring patterned responses throughout the dataset. The team went through several coding trials when the same segment of data was coded by all three researchers and then verified for consistency of codes used in a group. Inter-coder consistency was checked for and divergence in code interpretations was eliminated to the best of our ability.

## Results

### Self-reported Health

The majority of community participants reported their health as ‘*unsatisfactory*’ and/or ‘*poor.*’ During the community consultations ‘*good health*’ was never mentioned. Instead, respondents discussed a noticeable trend of declining health status. For example, a participant from Dnipropetrovsk shared the following:* “…in our whole nation we have lost a lot of healthy people in two wars, in the famine, and I can see that every generation is less healthy than the previous one. I compare myself to my Mom, and my health is worse than hers when she was my age, my daughter has a worse health than I did when I was her age, and the same I see with my grandchildren.*”

### Healthcare System

Respondents voiced concerns about unprofessional doctors that are poorly trained, lack the necessary expertise to properly care for patients, and/or to instill trust in the general population. As one participant from Vinnytsia explained: *“I think, first of all, that we don’t have enough good doctors. That’s because the majority of doctors now pay for their degree in medical school.”* Other important themes that emerged from the analysis related to the healthcare system included corruption in medical facilities, the general lack of medication and equipment in hospitals and clinics, as well as expensive and poor quality medication.

### Barriers to Accessing Healthcare

Results from the community consultations revealed that financial barriers in the form of both official and unofficial payments were among the top barriers in accessing healthcare. To illustrate, an individual from Simferopol described the following: “*I think we have the only barrier – it is money*.”

OOP payments were frequently discussed by participants, at all levels of the health system, ranging from visits with the family doctor, inpatient care, ambulatory and diagnostic services, and even care received from nursing staff. As one participant from Korolivka explained: *“You always have to pay there. To bribe doctors. I know this because my mother was in an in-patient hospital department with cancer and I had to pay a nurse for giving my mother injections. She was supposed to give them [the injections] because it was her job. But I had to pay her each time.”* The near fatal effects of this are apparent in the following excerpt from a Dnipropetrovsk participant:* “I saw an elderly woman some time ago in the hospital. We knew each other and started talking. She told me that they discovered she had a tumour and I asked her whether she will be going in for an operation. She replied that she is going to die because she has no money even for doing blood work before an operation.”*

Finally, a number of participants also talked about how the requirement of payment runs contrary to what should be the ethical and moral stance of doctors in relation to providing care:

*“… and often in an emergency department you see something terrible when a person comes with an acute pain or something like that and before they give this person any help, they ask first whether they have any money. If you don’t have money, they tell you to go home because we do not have anything to help you with. And this is scary because all these doctors gave an oath of Hippocrates. These people are not just unprofessional but simply immoral and unethical. That is what really makes me sad*” (participant from Simferopol).

### Quality of Life – Impacts on Health and Well-Being

The community consultations explored quality of life as a major theme among participants, including factors related to *satisfactory and unsatisfactory living*. Participants frequently named family life, children, and their employment as the main source of joy and satisfaction in their life. The following quotes illustrate what makes people happy: *“I have an interesting job and always do things I like. For example, I read books, embroider and bring up my children”* (participant from Korolivka); and *“If everything is going well in my family and at my work. If I wake up and go to work with joy, if there are good relationships among my colleagues”* (participant from Sumy).

However, the results revealed a greater focus on factors related to *unsatisfactory living.* The factors of dissatisfaction with life included: the polluted environment, political instability, economic hardships, and structural causes of unhealthy lifestyle behaviors. A participant from Kyiv said: *“Unemployment and environment negatively influence my life”*; and an individual from Simferopol shared: “*What makes my life unsatisfactory is difficult life circumstances, such as financial problems or issues at work. Sickness also makes my life unhappy*.” Another illustrative discourse from a Dnipropetrovsk resident: “*I think that another important factor that influences our life here is how much we work and get paid. First of all, I am never sure if tomorrow I am going to have work, and whether I will be paid for the work I do. Also, the wages I am getting do not correspond to the amount of work I do. And this is the same for many and many young working people in Dnipro.*”

### Environmental Pollution

Environmental issues (eg, air pollution, pesticides, poor quality of water and food) especially in large industrial centres such as Dnipropetrovsk and Donetsk, were a key factor related to unsatisfactory quality of life. For example, according to Dnipropetrovsk residents: *“...the amount of industrial factories in our city affects our health a lot. Every morning when I cross the bridge from the left bank of the river to the right I see all these pipes that pollute our air with dark smoke. And there is a constant cloud of pollution right above our city, which is a horrible sight to see*”; and “*I think everybody knows that people in the Dnipropetrovsk region are dying four times faster than people in other regions and are in general much sicker than other Ukrainians because of our polluted environment*.”

As one Donetsk resident put it: “*Overall, health here is probably one of the worst in Ukraine because of our pollution.”* Even participants from other regions referred to the polluted atmosphere in the Donetsk region when comparing the health of people in their own regions. For example, a resident of Lviv described the following:* “I want to compare Western and Eastern Ukraine, and say that our health is better than theirs because we don’t have so many harmful industries. I know two women from Luhansk region who come to the Carpathian Mountains every year for five months because they cannot live during the dry months in their home because they have asthma and they can’t breathe there.”* Individuals living in small towns (eg, Hola Prystan, Trostianets) and less industrialized cities (eg, Vinnytsia, Kherson) often mentioned how their health and overall well-being were positively influenced by cleaner environment and green spaces in their regions.

### Political Instability

The next key factor related to political instability and transition, and how the lack of democracy and corruption negatively influenced health. In the words of a participant from Kherson:

“*Political and economic transition in our country influences us, our generation of middle aged people is almost lost, during the last 30 years we are struggling to survive on a biological level. When we were educated in some sort of ideas and upbringing and then it was all taken away from us and did not give us anything instead, this all lead to stress. Many illnesses started out with Perestroika, because people could not find a new place in society for themselves. And such situation went on until now.*”

In Sumy, two participants had the following exchange, demonstrating how some individuals recognized a connection between quality of governance and quality of life: *“Our government is not democratic, and politics in our country makes our lives miserable … Let’s not talk about politics, let’s talk about health, ordinary people are not affected by politics … No, I think that the quality of life depends on the political regime, whether it is democratic or authoritarian.”* A respondent from a small town in Dzhankoj effectively summarized many of the sentiments shared by those participating in the community consultations by stating: “…*politically….we live in a very uncertain time and no wonder it affects our health*.”

### Economic Hardship

A third key factor identified was economic hardship, enjoined by unemployment, low salaries and high cost of living. Many participants continuously and directly connected these socio-economic determinants and resultant poverty with their health. For example, one person from the village of Busha said: “*because if I do not have a job but have to pay my bills and pay for my kid at school, then of course my blood pressure goes up and I have other health problems*.”

Respondents aged fifty or older shared their nostalgia of the former Soviet government when all their basic needs were met. The perception of significant disparities between the rich and the poor in present day Ukraine became an element of grievance for many who felt they had lived a portion of their life in a previously egalitarian country.

Uncertain work conditions, volatile job market and wages, overtime hours and unsatisfactory work conditions were often listed as underlying reasons for illnesses. As a participant from Dnipropetrovsk observed:* “I think that the ones who are most sick are the working young to middle age people who do not have time to go see a doctor because they work six days a week ten hours a day. When will they go to a clinic? Also there is no interest from the employer to have healthy employees, they do not pay you if you go on sick leave or try to check your health and need to go to a doctor.”*

Similarly, another Dnipropetrovsk participant commented: *“…people who are young now, the majority of them work illegally and their employers do not pay anything into the Pensions fund, so when I retire, I will have no money from that fund at all.*” An individual in Vinnytsia also shared the following: *“I think that the most vulnerable population now is in the 25 to 40 age range because they do not have their own apartments and they have no permanent jobs with a pension guarantee because all of them are working half legally.”* In this context, many of the older participants continued to compare contemporary Ukraine to its Soviet past, the latter being associated with the existence of a stronger, and more secure and safe public infrastructure that positively impacted the health of the population.

Political and economic instability were also linked to high levels of stress and lack of faith in the future, as evidenced in the following excerpts: “*Are there reasons to smile? People are so unsure about what will happen tomorrow that they have no reasons to smile. And this instability, uncertainty affects our health*” (participant from Dzankoj), *“…there are too many people who are depressed because of the general situation in the country, especially elderly people, they have no faith in tomorrow, no confidence in how they will live tomorrow*” (participant from Sumy), *“…all illnesses are starting because of stress – this is our problem because we have no assurance in the days to come, we have no certainty, and we are constantly worried about our lives. All these negative emotions make us sick*” (participant from Okhirmivka).

### Rural/Urban Divide

Rural/urban health was consistently and vigorously debated during the community consultations. On one hand, many participants believed that life in the villages with its physical work made people stronger and less stressed. Cleaner air and environment were mentioned as a positive determinant of health in rural areas. Furthermore, many discussed how rural residents were able to consume better quality produce and dairy products than urban dwellers who relied mainly on supermarket products. This is evidenced in the following excerpts: “*People in villages are stronger because we work more and are more active*” (participant from the village of Busha); “*People in the city have a worse health than us because they have more stress and the air is more polluted there*” (participant from the Pryvovchanske village); and: *“Life in the village is calm, paced, there is no constant noise like it is in the city, and this positively influences our mental health. When you come into town, you see that people there are always in a hurry, they are often stressed out. But our village life, its calm atmosphere is a big plus for our health. Everyone knows each other here, this is also good for our health*” (participant from Pryvovchanske).

Conversely, others argued that the health of villagers was more precarious because they experienced higher levels of unemployment and lacked community infrastructure. For example, two participants revealed the following: “*We have no places to spend our time, to have a rest. We do not have any art study groups, dancing classes or singing company to organize concerts. Youth has nothing to do here, that causes drinking alcohol, smoking*” (participant from the village of Korolivka), and *“One of the problems that we have in the village is that because of unemployment our youth drinks alcohol, smokes, and sometimes even takes drugs*” (participant from the village of Halchyn).

Concerning healthcare access by geographic location, participants from the community consultations reported that basic primary healthcare is often not available for rural residents. The lack of primary care clinics in many smaller villages, poor transportation to the nearest health centre, and poverty were named as the top reasons for inadequate access to healthcare among rural populations. In the words of a doctor from a district hospital in Hola Prystan: “*Yes, even sometimes we ask people who live in villages and who come to us being already very sick, we ask them ‘why did not you come earlier?,’ but they say that they did not have money for the ticket to come into town.*” One participant from the village of Korolivka effectively linked financial hardships in rural areas to health by describing the following: *“Everything is interconnected. If you don’t have money – you don’t eat good food, you can’t go to a health resort, buy warm clothes. The health is worse because of this. No job – no future. There are no prospects for the young generation.*”

Another issue raised by our participants was the detrimental effects of work migration within and outside the country. Conversations about the quality of life in rural areas often revolved around families being torn apart because one of the adults has to go seek employment either in the city or abroad, depending on the location of the village. One participant from Bania Lysovetska in the West of the country shared: *“It’s very hard for young families to survive these days. My husband and many other men have to go abroad and seek work there, because otherwise it’s impossible to live with 2000 hryvnia [Ukrainian currency] as a family, so now we see him on Skype or twice a year when he comes to visit. What kind of a family is that? It’s just a formality on paper.”*

### Health Behaviours

The majority of participants cited a huge concern about the lack of physical activity and high levels of alcohol and tobacco consumption – especially in relation to the declining health of the younger generation. As one participant from Donetsk pointed out:* “Young people today don’t move enough. They play too many video games. I know this from my own experience. That’s where so many problems with health come from, especially heart problems and problems with movement.”* Our findings further revealed how the situation is critically dire in villages where a pub or a corner store is often the only attraction or place for youth to spend free time. As a result, children as young as twelve years of age begin to engage in health-jeopardizing behaviours such as alcohol and tobacco consumption. As several respondents shared: “*They build night clubs, cafes, internet clubs. But not a single stadium or playground for children was built in our village. And children have no place to go*” (Halchyn participant) and *“We have no places to spend our time, to have a rest. We do not have any art study groups, dancing classes or singing company to organize concerts. Youth has nothing to do here, that causes drinking alcohol, smoking*” (Korolivka participant).

In this context, many pointed to the lack of publicly available, government sponsored infrastructure for exercise and healthy lifestyle promotion. A participant illustrated this problem by discussing the following: “*We also have no infrastructure to exercise and to lead an active lifestyle. Whatever clubs and fitness centres we used to have during the USSR are now either broken down or really expensive and people cannot take advantage of regular exercise*,” and another participant from Simferopol explained: “*There is a lack of alternative lifestyle to the stressed out life that young or middle age people could choose. The majority of people have no opportunity to exercise, to go dancing, hiking, like we used to do in the Soviet times. It is all too expensive now, all these swimming pools and fitness classes. There is no healthy leisure now. It is either too expensive or it is simply not there*.”

### Recommendations for Change

Participants in the community consultations were asked about what changes they would introduce to improve people’s health in their community and to the healthcare system. In terms of the healthcare system, respondents discussed the need to increase: (1) financing of the healthcare system, (2) the number of available and affordable healthcare services, and (3) physician salaries. Limiting OOP expenses, introducing non-financial incentives for health professionals and improving the quality of doctor education by eliminating corruption in medical schools were also emphasized: eg, “*Medical degrees should not be sold but only awarded to those who are dedicated to the profession and studied properly*” (participant form Trostianets), “*Medical schools need to be free and with no bribes, so that students who are really talented and hard-working get through and become doctors*” (participant from Zvenyhorodka).

Beyond the healthcare system, major themes for change included health promotion and prevention of disease, increased government regulations, and improved services in communities. For example, across all regions, participants highlighted the need to introduce and popularize health promotion in the general population, with a special emphasis on reaching the younger generation. One individual from Kherson proposed that: “*Our government should advertise a competition for the best project on how to improve the condition of public health in our region and for Ukraine as a whole*.” Many respondents emphasized the need to focus specifically on providing more information on the harmful effects of various substance addictions (while imposing limitations on availability and advertising of tobacco and alcohol) and sedentary lifestyles. Instead, the government and NGOs should encourage healthy behaviours and eating habits.

Better regulation over environmental degradation also figured prominently in the recommendations: eg, “*There should be stricter control of industries that pollute*” (Pryvovchanske participant); *“People and companies that pollute our environment must be accountable and pay a financial penalty”* (Kyiv participant); and *“We need to improve the sewage and water supply here”* (Zvenyhorodka participant). Further, the need for improvement in the garbage removal and recycling services, as well as improved public transportation were consistently highlighted across the consultations.

Participants had practical suggestions on how to create opportunities for more active and engaged lives that would deter the use of alcohol and tobacco, increase physical fitness and give citizens positive activities to engage in. Ideas ranged from the development of bicycle routes (*“…to create bicycle routes would be a great propaganda of healthy lifestyle. There is right now no possibility to ride a bicycle,”* participant in Cherkasy), repair and creation of sport and fitness facilities (*“if the city provided a land for building a sports park, then families would be more inclined to use it for recreation and that will alleviate the stress from everyday lives,”* participant from Sumy; and *“there are not enough playgrounds or outside gyms, which is negative for the development and health of our citizens. We need 40 or 50 of such places for our city,”* participant from Cherkasy), to organized community sport activities, (*“I suggest running camps like churches are doing already but the cities are unwilling to invest into – so that kids can just run around and play safely somewhere”* Lviv participant).

## Discussion

One of the limitations of the study is the absence of the demographic indicators for all of the consultation participants, which does not allow us to analyze the differences in opinions and the health and healthcare needs between males and females, young and old participants. The only two identifiers we collected consistently was the place of residence for each participant and gender. Some locations had very low numbers of attendees, which is another limitation of the consultations. The research team conducted the study autonomously from the Ministry of Health of Ukraine, which provided for a greater research freedom but at the same limited the dissemination of the findings among the policy-makers in the country. Two-thirds of participants in the study were female, introducing a potential bias in the selection of participants.

An important strength of this study is that community-based qualitative consultations, on this scale, have never been undertaken in Ukraine. Important previous work^20^ was conducted in 2003 in three regions, and focused on everyday understandings of health and factors influencing it. Our study consisted of a sample of Ukrainians across eleven regions, including urban and rural, encompassing the rich diversity of the population. Moreover, our study aimed at collecting information, using only community consultations, on a wider set of issues relating to health, as set out in the conceptual model of SDH.^26^ And finally, what made our study unique is that we engaged the public in discussions and recommendations for change, in terms of improving their lives and their health.

Our results are in-line with previous studies demonstrating a consistent trend of significantly worse self-reported health of the Ukrainian population. For example, in a 2002 study by Gilmore, McKee, and Rose, 25% of men and 43% of women rated their health as *poor* or *very poor*.^43^ Abbot and Wallace found similar results, where very few participants reported they had excellent health.^23^ Furthermore, our community consultations revealed geographic disparities with respect to how individuals and communities as a whole perceived their health. Environmental pollution, rural/urban economic and infrastructure divide, as well as work migration patterns were the main factors presented by our participants as important in affecting their health and well-being. This finding is comparable with a recent study which identified that residents of Central and Western regions of Ukraine reported the best-rated evaluation of their health, followed by residents of the South; the worst self-rated health evaluation in this study was reported by residents of Eastern regions (eg, Donetsk)^44^ similar to what was revealed in our community consultations. The majority of industries are located in the East of the country, and the participants residing in those areas gave a worse report on their quality of life and health in comparison to the participants from the rest of the country. And now this well-being divide will be further aggravated after the military conflict and the resulting anarchy in the Donetsk and Luhansk oblasts in the East of Ukraine. On the other hand, the Western and Northern regions in the country have more rural and remote are as with depressed economic situation, dilapidated healthcare centres, and no leisure opportunities. Participants in those locations expressed how these circumstances negatively affect their life and health causing some people to engage in unhealthy behaviours or leave their homes in search of work. In this respect, our research shows more aspects to the geographic disparities in self-rated health evaluations than the other study.^44^ The environmental benefits of rural life may be negated by the economic hardships and inaccessibility of healthcare in villages, whereas the reverse was expressed about the life in urban centres. It can be said, then, that participants thought of no region in Ukraine, which has overall advantageous circumstances for health and well-being.

Our study supports a growing body of knowledge that indicates the majority of the Ukrainian population reported high levels of dissatisfaction with the healthcare system and its related practices. Previous studies reported rates of dissatisfaction ranging from 75%^8^ to 82.6%.^3^ Similarly, a nationally representative survey conducted in 2010 revealed that less than one in five Ukrainians (17%) were satisfied with the work of the healthcare system.^45^ Another recent study reported that only 33% of the population is ‘*very or somewhat’* satisfied with the current healthcare system.^4^

Overall, specific concerns pertaining to the healthcare system were generally in-line with previous studies, bringing into sharp relief clear priority areas for system-wide reforms. Concerns about financial barriers are consistent with previous research where despite the fact that the Constitution of Ukraine (sec. 49) guarantees free access to healthcare, OOP payments create wide inequities, financial hardship, delays in or lack of any access to care.^4,5,38,46-48^ For example, 40% of Ukrainian payers borrow money or sell assets to cover hospital payments, and approximately 60% of those that need care simply forego services.^5^ Similarly, another study found that 55% of their household respondents agreed with the statement that: “*The amount of money that must be paid to doctors prevents me from using medical services.”*^49^ Previous studies have also shown how rural residents delayed seeking healthcare due to their inability to pay twice as often as urban residents.^39^

The reported mistrust in doctors discussed in our consultations aligns with a recent study reporting that only 22% of household respondents in polyclinics felt their physicians are well-trained, and that 50% of clinical patients had doubt or uncertainty regarding their physician diagnoses.^4^ A unique finding in our study – not widely covered in the literature to date – is the perception of healthcare users about pervasive corruption in medical schools, and the distribution of lucrative postings for newly graduated doctors.

Data emerging from the community consultations raised important issues for policy-makers to think beyond the scope of healthcare system in terms of reform priorities. Respondents did not spend as much time discussing issues with the healthcare system as they did the broader determinants affecting their lives and health. This in itself is an important finding since the concept and/or term ‘social determinants of health’ is largely unknown in Ukraine. Population health is in fact largely absent from the MoH key strategies, policies, and programs^18,50-53^ Experts have called for comprehensive SDH approach to deal with the health crisis in Ukraine.^34,43^ And as a recent WHO report on healthcare trends in the fSU has recently concluded, political leaders in these countries, including Ukraine, are ‘often not cognizant of the importance of SDH when considering policy.’^54,55^

The results from this study revealed, however, that the Ukrainian population itself recognizes the significant influence of determinants on health and well-being.

This is in line with the findings of Abbott et al,^21^ in terms of the findings around the environment and finances. These are important findings as they reveal how many issues remain salient in the Ukrainian context in relation to health, and how in many ways, little has changed in a 10 year period of time. But the findings were also broader, including for instance, issues of politics, rurality, health of children and youth. For example, there is virtually no attention paid in current policies to rural health needs in Ukraine.^55^ There is only a small section devoted to rural health and healthcare services in the State Target Program on the development of Ukrainian rural areas for the period till 2015, which does not contain specific policy steps to improve rural health and healthcare access.^56^ In 2011, the Cabinet came out with the National Conception for Healthcare Reform, which acknowledged that the access to healthcare is ‘distributed disproportionately between rural and urban territories,’ but did not suggest any solutions to this problem.^57^ This lack of attention to the health needs of rural populations is especially concerning since the government analysis concluded that rural areas experience a health and socio-economic crisis leading to the die-off of villages.^56^

Health promoting needs of populations is also an area of neglect. Our study illustrated the overwhelming concern for children and youth. Other research is starting to show how this is a population requiring specific consideration. For instance, a 2012 international report by the WHO on health behaviours in school-aged children in Europe reported significant drops in physical activity levels for the Ukrainian children between the ages of 11 and 15, with lower drops for young girls than boys. The report globally ranked Ukraine as number one in reported sedentary behaviours such as watching television.^58^ Overall, the health of children and youth should become more of a policy priority.

Of course, given the current situation of conflict and uncertainty, political change and economic transitions will take a considerable amount of time. Addressing such structural determinants of health will take sustained efforts by current and future governments. However, as the following excerpt underscores, the public in our study voiced great skepticism about those in power: ‘*Even this conversation [eg, community consultation] – it’s not going to go anywhere. Our government…they don’t care*.’ The distrust of government officials in post-Communist countries is a well-documented fact^59,60^ and something that politicians need to begin to address as part of any reform effort. It is also clear from what is currently happening in Ukraine, however, that the government has limited power to change or improve the political and economic situation without the assistance of the broader international community.

At the same time, our study shows that the Ukrainian public wants to take action on the SDH. This is a noteworthy departure from 2003 findings^26^ where qualitative research showed that the population of Ukraine is at a loss to know how to change the situation. Moreover, our informants revealed that they have *realistic,* and in many instances, very *practical* suggestions of where such action should start (eg, government regulations on environmental protection, improving municipal services and programs, developing effective health promotion for the population through media and schools, and limiting the availability of tobacco and alcohol).

## Conclusion

Fully understanding health crises in any country requires a broad base of evidence. In this study, we contributed to this base of knowledge through community consultations that were focused on capturing the myriad of factors that affect health inequities and which should all be targeted in terms of policy development and intervention in the Ukrainian context. Our research also demonstrates through the perceptions of the affected population that much of what contributes to Ukraine’s health crisis is grounded in the SDH and must in fact involve sectors including but not limited to health. Future research could investigate some of factors reported here in more depth, and choosing to focus on a smaller number of distinct regions of the current. At the same time, integrating the findings of our research in current reform efforts in Ukraine would not only ensure health and healthcare policies tailored to the needs of the population but also follow international standards of best practice – health in all policies – which requires coordination across different social, environment and economic sectors to identify and take action on the root causes of health inequities. For a country that faces an uncertain future, such information is vital for improving health, and fundamental to human, social and economic development to ensure future reform efforts include, but are not narrowly limited to health system improvements.^61^

## Acknowledgements

The research was funded by the Canadian Institutes of Health Research, Ottawa, ON, Canada.

## Ethical issues

The Office of Research Ethics at Simon Fraser University, Burnaby, BC, Canada.

## Competing interests

The authors declare that they have no competing interests.

## Authors’ contributions

The leading author is OH, who was the Principal Investigator of this research project. The other three authors have made equal contributions to the collection, coding, and analysis of data as well as writing and reviewing the manuscript.

## Endnote


[1] For more detailed discussions on the Ukrainian healthcare system see: Peabody et al,^4^ Hankivsky and Vorobyova^35^ and Tarantino et al.^17^


## Authors’ affiliations

^1^London School of Hygiene and Tropical Medicine, London, UK. ^2^School of Public Policy, Simon Fraser University, Vancouver, BC, Canada. ^3^Institute for Intersectionality Research and Policy, Simon Fraser University, Vancouver, BC, Canada. ^4^Institute of Population Health, University of Ottawa, Ottawa, ON, Canada.

## Key messages

**Implications for policy makers**

- Community consultations conducted in Ukraine in 2012 revealed public’s awareness of how wider social determinants – beyond the healthcare system – shape the quality of their health.
- Citizens are eager to provide their input into government decisions that affect their health; it is recommended that policy-makers consult with the broader public on how their health and healthcare experiences could be improved.
- This study brings to attention of policy-makers the importance of various research methods and different forms of evidence; it specifically underscores the importance of community consultations.
- It can also aid policy-makers and healthcare professionals to identify important areas of perceived healthcare needs for developing effective programs and policies.
- The health crisis in Ukraine cannot be solved by means of reforming only the delivery of healthcare services.
- In addition to modernizing the delivery of health services, the reform of the health sector in Ukraine should include actions in the areas of healthy living promotion, alcohol and tobacco control, and environmental protection.

**Implications for public**

Community consultations are one of the effective vehicles to educate policy-makers about the public’s lived experiences, specifically in the context of healthcare reforms. Implications for the public in particular include: public development (by encouraging a broad range of participation) improving participants’ knowledge of the subject area and increasing awareness of the complexities of health. This study revealed that Ukrainian citizens are keenly aware of the dire health situation in the country and the need to modernize the healthcare system. At the same time, the participants shared their understanding of how broader socio-economic determinants (eg, political instability, poverty, the poor quality of water and air, availability of tobacco) influence the decline in health status. Importantly, participants were actively engaged in using their knowledge and experiences to develop recommendations and solutions to the health crisis in Ukraine, a process that can improve community cohesion and empowerment.
